# Electrochemistry of Azobenzenes and Its Potential for Energy Storage

**DOI:** 10.1021/acs.joc.5c00315

**Published:** 2025-04-14

**Authors:** Dominic Schatz, Hermann A. Wegner

**Affiliations:** † Institute of Organic Chemistry, Justus Liebig University, Heinrich-Buff-Ring 17, 35392 Gießen, Germany; ‡ Center of Materials Research (ZfM/LaMa), Justus Liebig University, Heinrich-Buff-Ring 16, 35392 Gießen, Germany

## Abstract

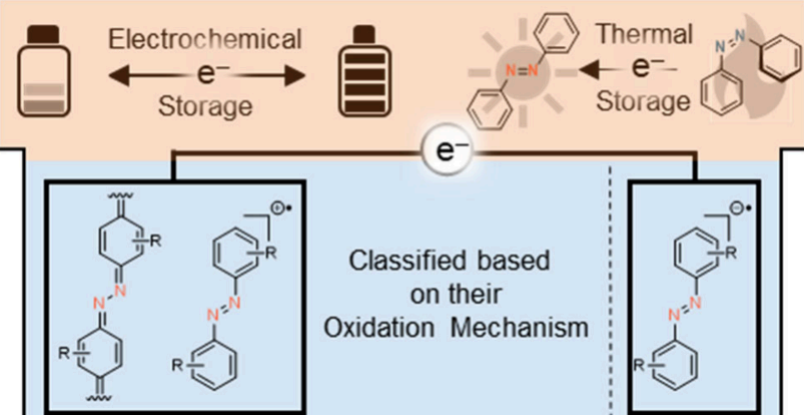

Azobenzenes are promising materials for energy storage due to their reversible photoisomerization and redox properties. Given the critical role of redox behavior in the latter application, an investigation of their redox processes is essential. We propose a classification of azobenzenes into two categories: *benzenoid*-type and *quinoid*-type, based on the mechanism of their oxidation. *Benzenoid*-type compounds have been extensively studied due to their reversible reduction. *Quinoid*-type compounds exhibit oxidative and reductive versatility, making them promising for further research in energy storage.

Undoubtedly, azobenzenes (AB) belong to a class of privileged structures, which have extensively been studied since their discovery by Mitscherlich in the middle of the 19th century.^1^ The versatile applications in many different fields can be traced back to mainly three properties: Their bright coloration,^2^ their reversible photoisomerization,^3^ and their redox chemistry.^4^ By focusing on their photophysical properties, AB can be divided into three different classes depending on their n-π* and π-π* transitions, and various overviews deal with the respective dye or photoswitch properties of these classes.^5^ Generally, the incorporation of nitrogen atoms into a molecular scaffold significantly alters the electronic nature.^6^ For example, the first reduction wave of AB is more facile by 800 mV in comparison to stilbene (−1.4 V vs −2.2 V, respectively vs saturated calomel electrode).^7,8^ Additionally, π-delocalization changes the electronic behavior.^9^ Therefore, AB as a nitrogen-containing π-extended compound offers interesting opportunities to control its redox chemistry via structural variation. ABs show distinct reductive and oxidative redox performance and can be divided into two categories, depending on the mechanism of their oxidation. Most AB show irreversible oxidation, which we assign to the first, the *benzenoid*-type category. We categorize AB that can form a quinoidal system upon oxidation into the second, *quinoid*-type group (Figure 1). Herein we want to introduce the underlying redox properties of both of these classes and showcase current applications based on their respective reductive and oxidative behavior. A comprehensive assessment and comparison of the performances in AB-based battery solutions can be found in a review by Shimizu, Tanifuji and Yoshikawa.^4^

**Figure 1:**
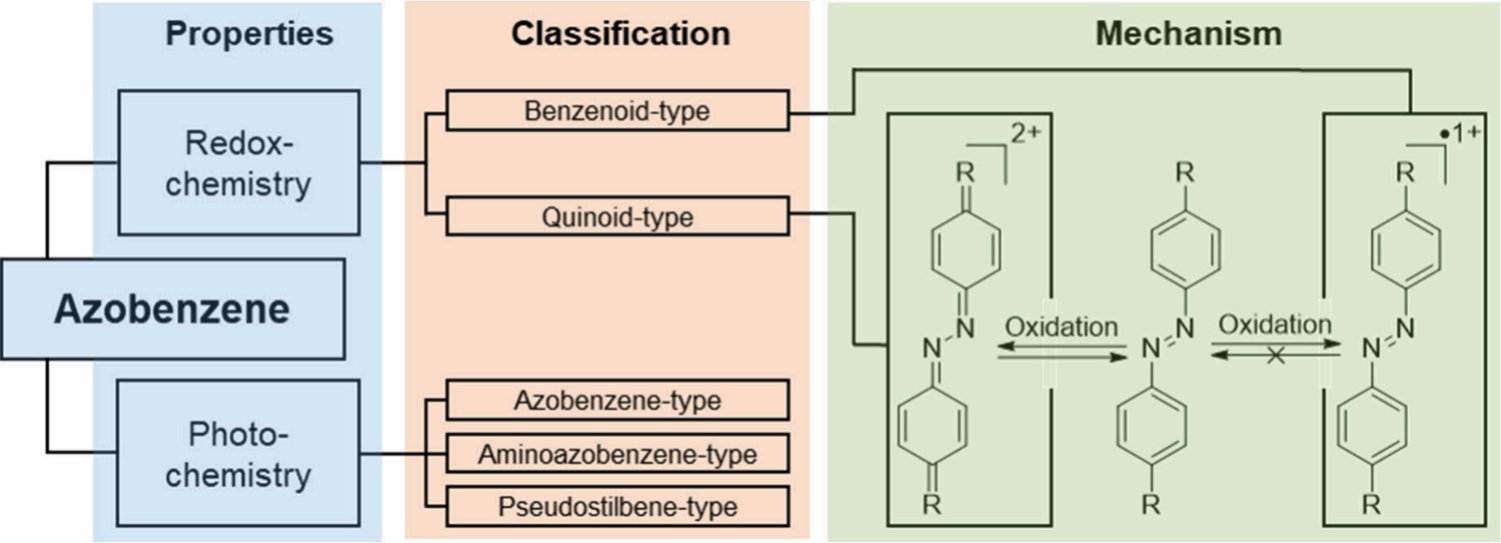
Classification of AB in *benzenoid*-type and *quinoid*-type, depending on the reversibility and mechanism of the oxidation process. The formed radical cation of *benzenoid* compounds decomposes rapidly, while the benzoquinone imine structure of *quinoid* AB is stable.

## *Benzenoid*-Type ABs

The redox chemistry of *benzenoid*-type AB was already investigated by Schlenk and Wittig, who studied the addition of metal to different double bonds. Addition of sodium to pristine AB **1** results in a dark purple solid, which, after quenching with water, yields equimolar hydrazobenzene and neutral AB.^10^ The result was, therefore, not the expected 1e^–^ reduction product as a monosodium radical anion but a mixture of neutral compound and the 2e^–^ reduction product. Addition of 2 equiv of methyllithium, on the other hand, formed bis-lithiated hydrazobenzene, which formed a radical anion after addition of a second equivalent of AB.^11^

**Figure 2:**
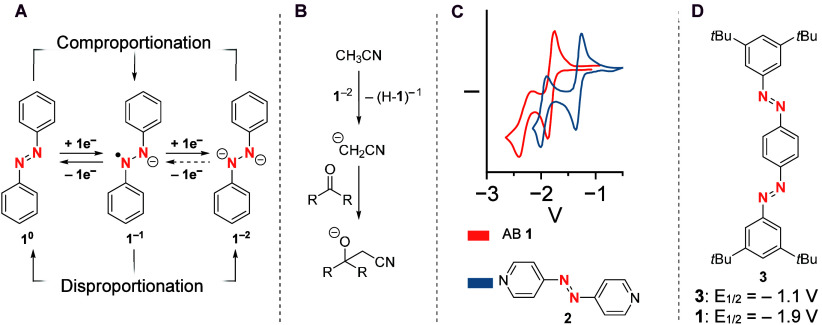
A) The two consecutive reductions of AB to the anion radical and the bis-deprotonated hydrazine, and their interconversion via comproportionation and disproportionation reactions. B) Electrogenerated AB **1**^–2^ base yields a reactive cyanomethyl anion. C) Cyclic voltammogram (CV) measured with ferrocene (Fc) and referenced vs Fc/Fc^+^ of pristine azobenzene (**1**) and 4,4′-azopyridine (**2**) highlighting the pseudoreversible second reduction of the electron poor derivative.^16,21^ Copyright © 2024, American Chemical Society. D) Structure of bis-AB **3** and its reduction potential vs Fc/Fc^+^ compared to the reduction potential of AB **1**.^22,23^

This comproportionation of deprotonated hydrazobenzene was further studied and showed a strong dependence on the employed countercation, as well as an electron paramagnetic resonance (EPR) signal that is influenced by hyperfine splitting of the alkali metal.^12^ Even just the mixture of AB and hydrazobenzene yields a radicaloid species observable by EPR, indicating an electron transfer between the compounds with oxidation state −2 and −1 (Figure 2, A).^13^ In electrochemical experiments, AB behaves differently depending on solvent, electrolyte, and electrode material. During polarography in aprotic, polar solvents, AB is reduced in two consecutive 1e^–^ waves.^14^ The first wave produces the radical anion, as observed by EPR and UV–vis measurements, and the second wave produces the bis-anion. Under most conditions, the first wave appears reversible on the time-scale of the electrochemical measurements, while the second wave appears to be irreversible. The irreversibility of the second wave can be explained with the aforementioned comproportionation, as well as the significant basicity of the electron-rich bis-anion, leading to side reactions (Figure 2, B).^15^ An example of this is the semireversible second 1e^–^ reduction of electron poor 4,4′-azopyridine **15**, which is more reversible then for the pristine AB **1** (Figure 2, C).^16^ Elucidation of the structure and geometry of the reduced AB species was obtained by single crystal XRD. In earlier studies the electron transfer from two ABs to Re(0) yielded a paramagnetic Re(II) complex of two AB radicals.^17^ In the obtained crystal, an elongation of the N–N bond in comparison to pristine AB was observed as well as a shift in the ν(N–N) IR band to lower wavenumbers, indicating a decrease in the double bond character of the radical in comparison to the neutral compound. The observed bond elongation and the obtainable EPR signals are in agreement with the location of the radical on and over the azo nitrogen atoms. Similar results have been obtained in osmium^18^ and ruthenium^19^ complexes, or with imidazole based AB.^20^ In 2020, the free radical and bis-anion of an AB derivative were isolated after reduction with alkali metals in the presence of crown ether (N–N bond lengths of neutral, radical, and bis-anion are 1.252 1.326, and 1.391 Å, respectively).^25^ Due to the free N–N bond in these anions, they were able to coordinate and activate CO_2_, yielding oxalic acid. A radical anion structure based on a quinoidal system is also proposed.^26^

**Figure 3:**
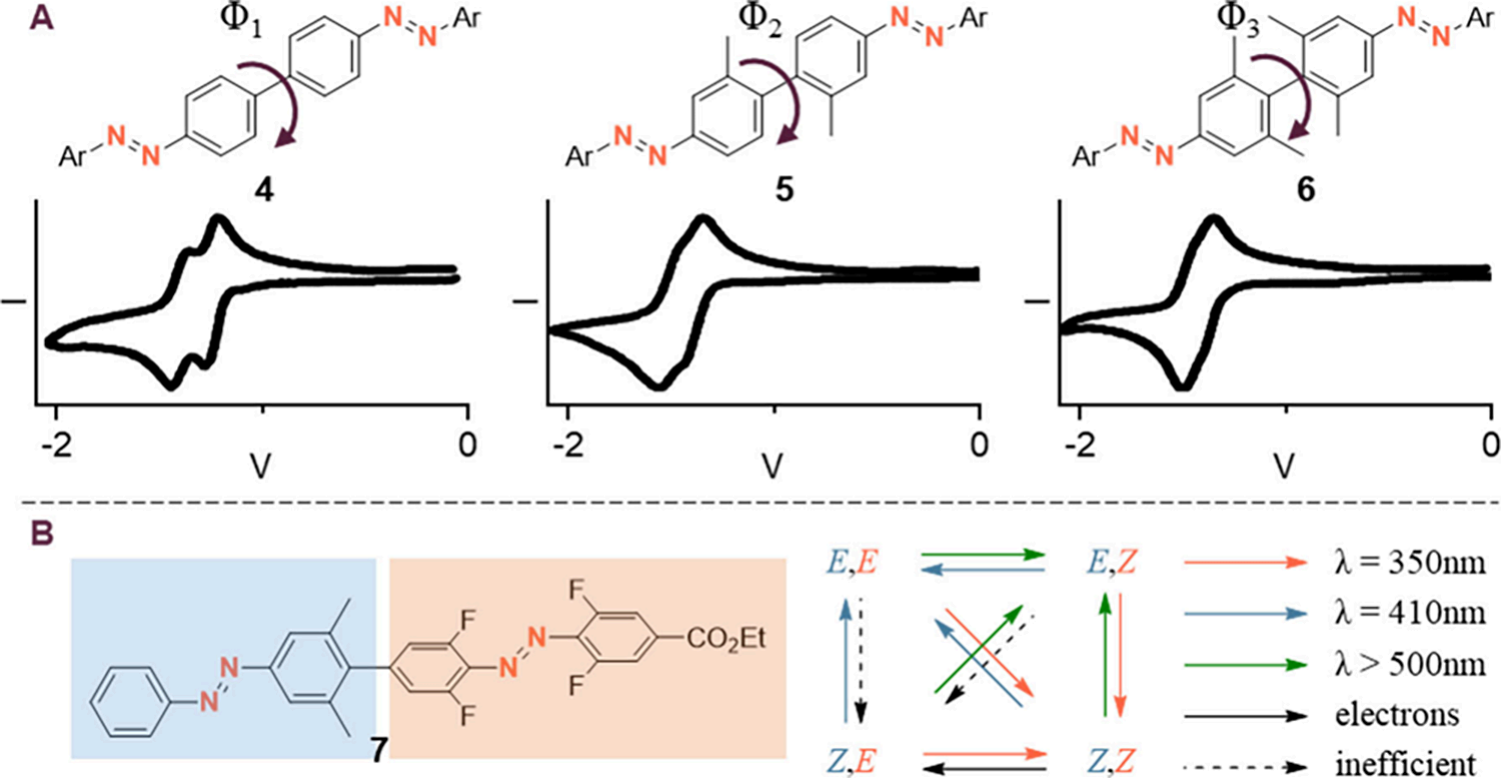
A) Dependence of the peak-to-peak separation in the CV of bis-azobenzenes **4**–**6** based on the dihedral angle between both photoswitches. CVs are referenced vs Fc/Fc^+^ The higher the dihedral angle, the smaller the peak separation, as the independent redox behavior increases with increasing angle.^23^ Copyright © 2011, American Chemical Society. B) Asymmetric bis-azobenzene **7** and the pathways to achieve orthogonal switching, with the different reduction potential allowing selective (*Z*) to (*E*) switching of the more electron poor switch.^24^

By extending the π system in *para* connected bis-AB, their reduction proceeds more facilely in comparison to pristine AB (Figure 2, D).^23^ The *para* bis-AB **3** shows two clearly distinct reversible 1e^–^ reductions for each azo unit that are separated by 0.5 V. This clearly shows the strong electronic communication in *para* ABs, as also demonstrated by ultrafast isomerization dynamic experiments.^27^ If the azo units are separated by a biphenyl linker, the first two 1e^–^ reductions proceed at more similar potentials, as they behave more independently of each other. Increasing the dihedral angle between the biphenyl moieties (from **4** to **6**) by adding steric bulk results in one indistinguishable redox wave for both redox processes (Figure 3, A). This charge delocalization of quasi planar *para*-bridged oligo-AB has also been visualized by EPR.^28^ The observed delocalization is smaller than that in the structurally akin stilbene compounds, which is attributed to the large degree of spin density on the nitrogen bridge. Furthermore, *para* biphenylene connected asymmetric bis-AB **7** was able to switch orthogonally by a mixture of photochemical and electrochemical stimuli (Figure 3, B).^24^ The reason for that is the discussed loss of electronic communication due to rotationally hindered biphenyl bridges as well as two clearly different redox potentials due to different substitution on the AB rings. The oxidative behavior of *benzenoid*-type compounds is less explored, mainly due to the reason that most AB derivatives show irreversible and destructive oxidation behavior.^29^ Nevertheless, an EPR spectrum of AB **1** was obtained after bombardment with γ-rays of AB in a frozen CFCl_3_ matrix,^30^ and later in a frozen cryofluorane matrix at 77 K.^31^ The formed radical cation can also be observed in the isomerization of a bulk solution of AB after addition of oxidation reagent, albeit with destructive behavior.^29^

## *Quinoid*-Type ABs

**Figure 4:**
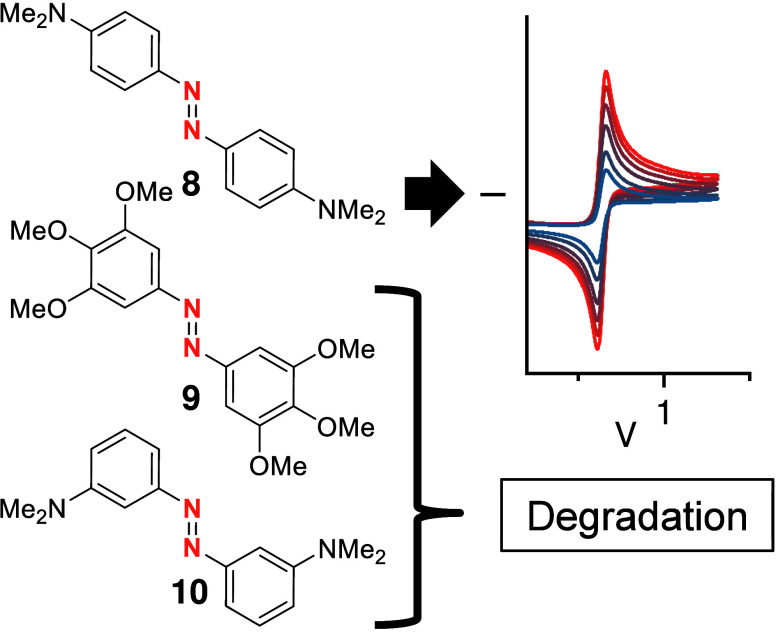
Reversible oxidation of 4,4′-dimethylamino AB **8** is based on the formed quinoidal oxidized species, as neither the electron-rich hexamethoxy AB **9** nor the 3,3′-dialkylamino AB **10** show reversibility.^35,36^ CV is measured with decamethylferrocene (Fc*) and referenced vs Fc*/Fc*^+^. Copyright © 2024 The Author(s). Angewandte Chemie International Edition published by Wiley-VCH GmbH.

The second class of redox-active AB consists of bis *ortho*- and *para*-connected amino- or hydroxyl-substituted compounds. They are distinct from the first class, as they show a stable 2e^–^ oxidized quinoidal structure, that allows reversible oxidative behavior.^32,33^ Although earlier studies observed only a 1e^–^ oxidation of 4,4′-diamino ABs by electron poor quinodimethanes, forming the corresponding 1:1 salts,^34^ more recent electrochemical studies and redox titrations by the group of Hecht confirmed the 2e^–^ oxidation, of, for example, AB **8** (Figure 4).^35^ Spectro-electro measurements demonstrated a red-shifted intermediate, which hints to the formation of an radical cation by 1e^–^ oxidation, followed by an fast consecutive 1e^–^ oxidation to the quinoidal system.^35,36^ 3,3′,4,4′,5,5′-Hexamethoxy AB **9** and 3,3′-dimethylamino AB **10** were prepared to demonstrate that the origin of the reversibility lies in the formation of the quinoidal structure, and not just on the electron-density of these compounds, as both showed oxidative degradation (Figure 4).^35^

**Figure 5:**
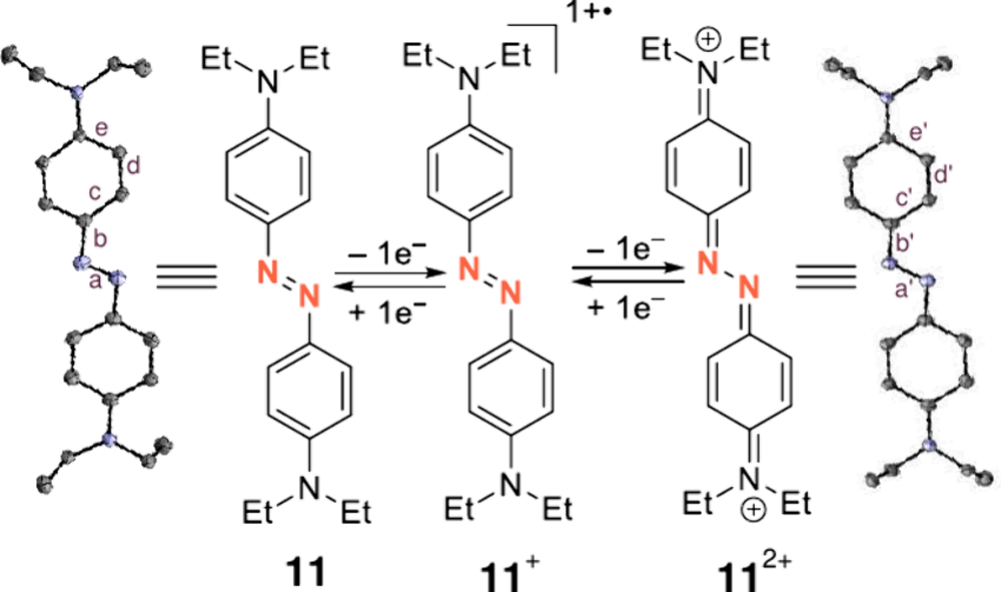
Oxidation of *quinoidal* AB **11** (CCDC: 1000644)^37^ via a cation radical, observable by spectro-electro measurements, and the formed stable 2e- oxidized species. Geometry of the oxidized compound was confirmed by single crystal XRD (CCDC: 2247535).^36^ Solid state structures are depicted without solvent molecules and anions. Bond lengths in Å: a = 1.266, a′ = 1.363, b = 1.423, b′ = 1.321, c = 1.385, c′ = 1.442, d = 1.382, d′ = 1.346, e = 1.417, e′ = 1.450.

The stabilizing two electron process was rationalized with the formation of a benzoquinone imine azine structure by comparing ^1^H NMR signals before and after oxidation of AB containing hydroxyl groups at both *para* positions.^33^ The obtained signals are shifted downfield in contrast to the neutral compound and are different from the signals of the corresponding azoxy compound, eliminating azoxy compounds as possible oxidation products. To get further evidence into the structure of the oxidized species and the mechanisms of the reversible oxidation, we crystallized the product **11**^2+^ obtained after oxidation of 4,4′-diethylamino AB **11** with 2 equiv of NOBF_4_ (Figure 5).^36^ The obtained solid-state structure revealed that the predicted quinoidal structure is valid. A bond elongation of the N–N bond, a shortening of the N–C_aryl_ bond, and a bond alternation in the ring systems all indicate quinoidal character over the whole AB core. Furthermore, calculation of the δr^38^ value, a value used for quantification of quinoidal character, of 0.100 Å verifies fully quinoidal rings.

**Figure 6:**
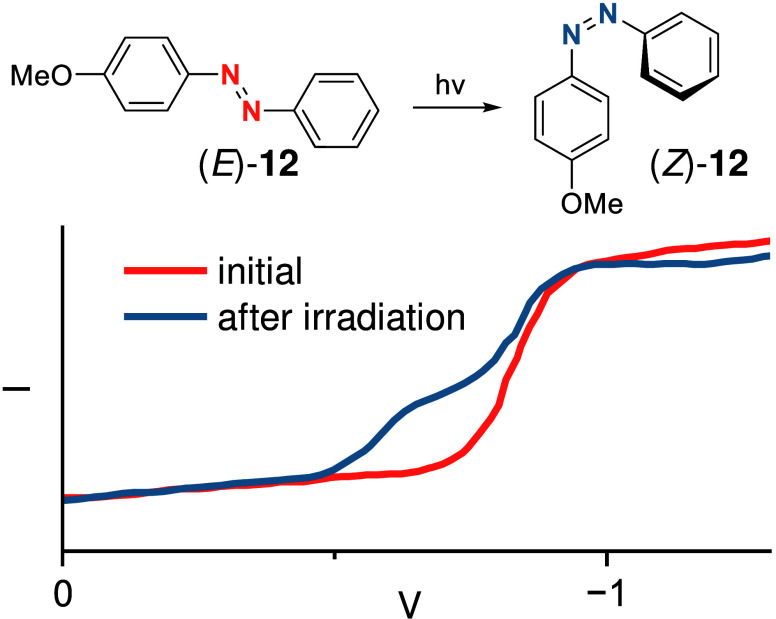
Difference between the polarograms of *para* methoxy AB **12** in 70% aqueous dioxane before and after photoisomerization. The new signal corresponding to the (*Z*)-isomer is shifted to more positive potentials and corresponds to the polarogram of a pure (*Z*)-isomer sample.^39^ Copyright © 1974, American Chemical Society.

One of the most prominent features of AB is the ability to isomerize from the stable (*E*)- to the metastable (*Z*)-isomer by irradiation with the appropriate wavelength. Isomerization leads to changes in properties, such as basicity, end-to-end distance, solubility, and dipole moment, and this raises the question of whether the electrochemical properties are also affected. Unfortunately, *quinoid* ABs usually show short half-lives in comparison to their *benzenoid*-type analogs, so that mostly *benzenoid*-type (*Z*)-switches have been studied electrochemically.^5^ Polarograms of *para*-methoxy AB **12** in aqueous dioxane electrolyte gave an additional signal after irradiation of the sample with UV light (Figure 6).^39^ The height ratio of both peaks was dependent on the irradiation duration, but the sum stayed constant, which correlates with photoisomerization. The metastable isomer can be more easily reduced by nearly 200 mV. The result of the observed 2e^–^ reduction is the same starting from the (*E*)- or (*Z*)-isomer. If similar experiments are carried out in DMF solutions, no difference in the redox potential of both isomers at room temperature is observed.^40^ Cooling the electrolyte to −22 °C and using very high sweep rates results in distinguishable cyclic voltammograms of (*E*)- and (*Z*)-AB. Photomodulated voltammetry, a technique typically used for elusive radical species,^41,42^ of in situ generated (*Z*)-AB in a variety of organic solvents revealed also a difference between both isomers.^43^

The underlying challenge of electrochemical analysis of the (*Z*)-isomer is the formation of the anion radical, which has an activation energy for the isomerization of only around 8 kJ mol^–1^.^40^ Pairing the fast kinetic constant with a radical transfer to (*Z*)-AB in the vicinity of the electrochemically generated radical, the metastable isomer can only be observed in voltammograms if the sweep rate is higher than the rate of isomerization.

**Figure 7:**
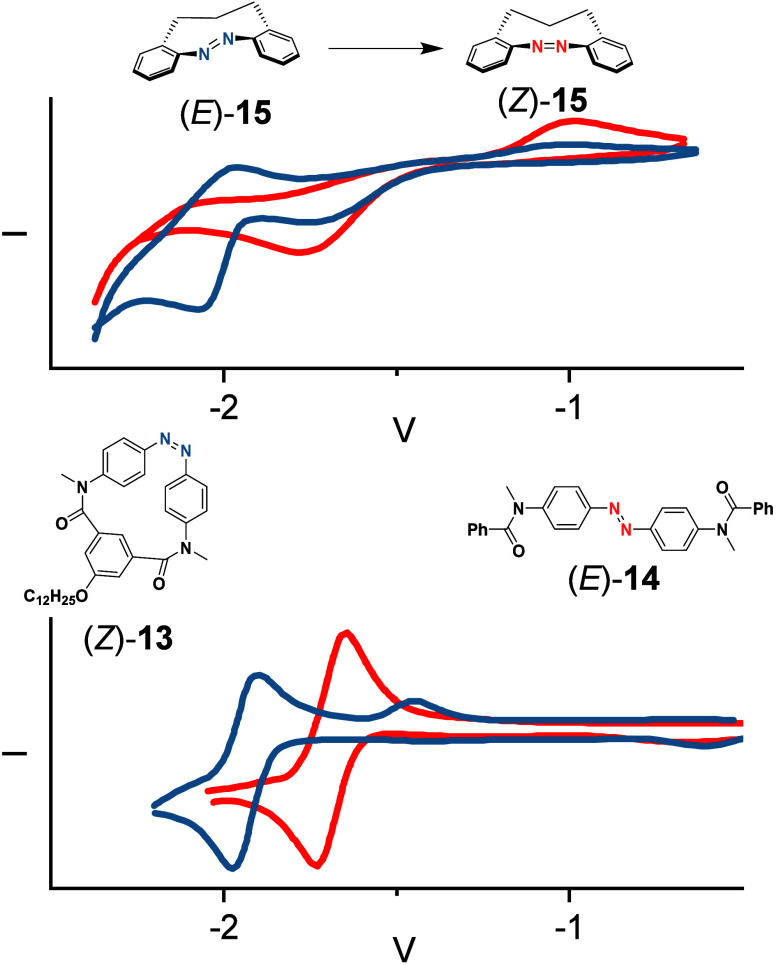
Top: Herges’ diazonines **15** where the (*Z*)-isomer (thermodynamically stable, red) and (*E*)-isomer (metastable, blue) show different redox potentials observable by CV measurements, due to the stability of the formed radical anion that only undergoes slow radical transfer.^44^ Copyright © 2023 The Authors. Chemistry - A European Journal published by Wiley-VCH GmbH. Bottom: A cyclic derivative **13** of (*E*)-AB **14** (blue) as a model for the (*Z*)-isomer with different redox potentials. (*Z*)-AB **14** isomerizes due to fast radical transfer on the time scale of the CV experiment.^22^ CVs are referenced to Fc/Fc^+^. Copyright © 2017, American Chemical Society.

Nevertheless, Hecht and Herges found ways to demonstrate the change in the redox potential upon isomerization. Hecht prepared a cyclic AB (**13**) and compared it to an analogous linear compound (**14**).^22^ He observed no difference of the (*E*)- and (*Z*)-isomer of **14** in the voltammograms, while the cyclic derivative showed a difference of around 250 mV to more negative potentials for the 1e^–^ reduction (Figure 7, bottom).

The former observation was rationalized with the catalytic back isomerization during scanning. By addition of a small amount of generated AB anion radical to a bulk solution containing (*Z*)-AB, fast isomerization to (*E*)-AB was observed. Using a spectro-electrochemical setup, the fast isomerization of the (*Z*)-isomer to its stable isomer was observable by its change in the UV–vis spectra during cyclic voltammetry, even before the onset of the (*E*)-isomer redox potential was reached. Herges investigated other cyclic diazo derivatives, the 1,2-diazocines. Their redox behavior is strongly dependent on the length of their alkyl chains, although all show the expected 1e^–^ reduction of azo compounds.^44^ If the linker is increased to a C3-chain (diazonine **15**), yielding a nine member central cycle, reduction of the metastable (*E*)-isomer does not yield the (*Z*)-isomer in a catalytic fashion, while C1- and C2-chains isomerize upon reduction. The obtained redox potentials of the (*E*)-isomer are shifted 130 mV to more positive potentials, demonstrating a clear difference between both photoisomers (Figure 7, top).

## Storage Applications

**Figure 8:**
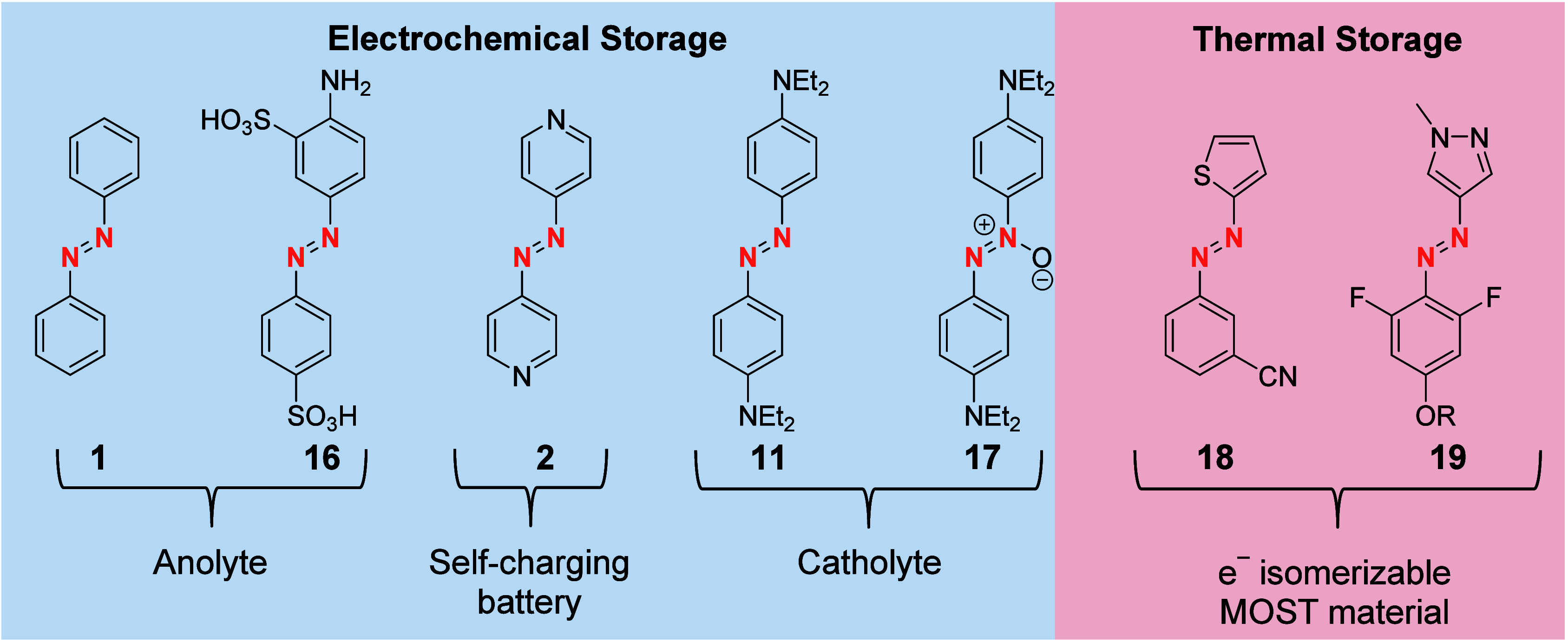
Some derivatives of AB applied in energy storage solutions. Most of these compounds (**1**, **16**, **2**, **18**, **19**) show *benzenoid*-type redox chemistry.

**Figure 9:**
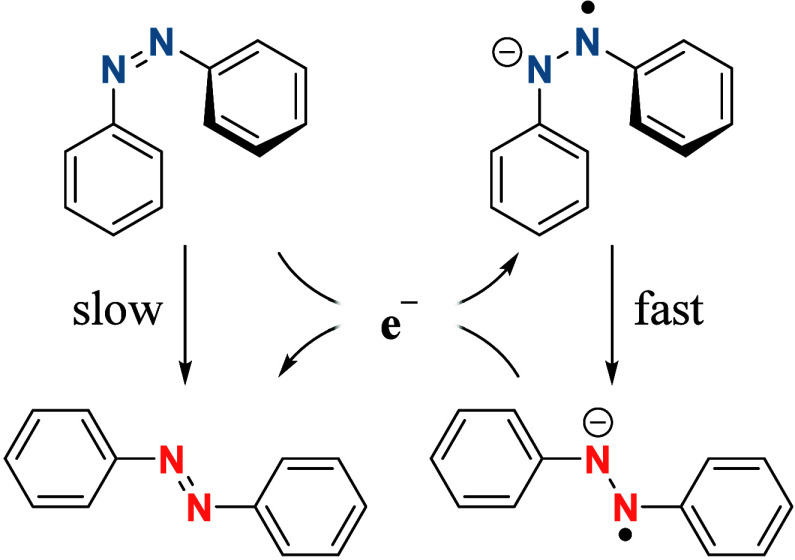
Proposed reaction mechanism for the electrocatalytic (*Z*)- to (*E*)-isomerization of ABs, that is based on a smaller isomerization energy barrier for the radical anion compared to the neutral compound. Intermolecular electron transfer from isomerized (*E*)- to (*Z*)-AB makes this process catalytic in electrons.

Both classes of AB have found their way into electrochemical or thermal energy storage applications (Figure 8). Due to the reversible reduction of most AB from the *benzenoid*-type class, as well as only marginal solubility in employed solvents, they show great performance as electrodes in metal-based batteries.^45^ Thanks to a relatively easy access to derivatives, their properties can be fine-tuned to succeed in aqueous (compound **16**) or organic (compound **1**) applications.^46^ An additional battery technology that employs *benzenoid*-type compounds is redox-flow batteries, where their reversibility allows them to be employed as the active anolyte.^47^ Moreover, the catalytic isomerization upon addition of either oxidant or reductant to produce radical cation or anion, respectively, can be used in molecular organic solar thermal storage (MOST). For MOST, a molecular photoswitch like AB absorbs light and transforms this energy into chemical energy.^48^ The thereby stored thermal energy can be later released and used. For an efficient MOST material, the heat release needs to be triggered to allow for on-demand applications. The redox chemistry of the employed photoswitches is perhaps one of the most efficient and promising triggers. The lowering of the isomerization barrier by radical formation, and the radical transfer to other photoswitches results in the complete isomerization of the (*Z*)-isomer (Figure 9).

Hereby, the reductive pathway via anion radicals, as well as an oxidative pathway via the radical cation with hetero AB similar to azothiophene **18** have been explored.^29,49^

The oxidative pathway shows lower cyclability due to the instability of the formed radical in comparison to that of the reduced species. By derivatization of azoheteroarenes with ionic side chains (compound **19**), an efficient electrocatalytic heat release can be realized even in the condensed phase.^50^

*Quinoid* ABs, on the other hand, can be employed as the catholyte in redox flow batteries.^36^ Hereby a larger capacity decay is observed over multiple cycles, possibly due to the instability of intermediate radical cations. Full symmetric RFB based on the oxidation and reduction of a single *quinoid* AB could not yet been established, as the reduction appears to be not reversible on the time scale of charge/discharge cycles. This might be due to the electron-rich nature of the employed 4,4′-diethylamino AB (**11**). Similar results were obtained by employing the structurally related *para*-amino azoxybenzenes (compound **16**) as the catholyte.^51^

Although the redox chemistry of AB has been known for quite some time, its applications in electrical and thermal energy storage are currently studied, mostly as electrodes in metal batteries, as the redox active material in flow batteries, or as a release trigger for MOST materials. In these cases, most employed structures belong to the class of *benzenoid*-type ABs due to their reversible reductive behavior. The potential of the reversible oxidation of *quinoid* AB has yet only been demonstrated as redox active materials in catholytes although we hope that further research will increase the stability and reversibility of *quinoid* AB for comparable energy storage technologies.

## Conclusion

The redox chemistry of AB offers multiple features for applications. The structure of the intermediated radical can be used to catalytically induce a thermal heat release in MOST energy storage systems, and the reversibility of this redox process can be used for electrochemical energy storage in metal-based batteries or in redox-flow batteries. Nevertheless, the high electron density in the reduced form of AB needs to be controlled to increase electrochemical stability, as side reactions with solvent or electrophiles are possible and, in the case of electron generated bases, even desired.

The oxidative behavior of AB, on the other hand, is a less established field, possibly due to the instability of formed radical cations, and only the further formation of a quinoidal oxidation product after a two electron process makes the oxidative side reversible. As the quinone formation results in a different oxidation mechanism, we classified AB based on their redox properties into *benzenoid* and *quinoid* like classes. With this categorization based on the underlying oxidation mechanism, we hope to increase the visibility of the versatility and applicability of AB redox chemistry and to facilitate and accelerate further research endeavors.

## Data Availability

The data underlying this study are available in the published article. Data for Figures 2, 3, 4, 6 and 7 have been extracted from their respective publications using https://automeris.io/.

## Abbreviations

AB: azobenzene
EPR: electron paramagnetic resonance
CV: cyclic voltammetry or cyclic voltammogram
MOST: molecular organic solar thermal storage
